# Uptake of the lateral flow urine LAM test in Europe and Central Asia

**DOI:** 10.5588/ijtld.21.0656

**Published:** 2022-09-01

**Authors:** C. Kraef, A. Yedilbayev, N. Seguy, A. Bentzon, D. Podlekareva, D. M. Cirillo, M. J. van der Werf, O. Kirk

**Affiliations:** 1 Centre of Excellence for Health, Immunity and Infections (CHIP), University of Copenhagen, Copenhagen, Denmark; 2 Department of Infectious Disease, Rigshospitalet, University of Copenhagen, Copenhagen, Denmark; 3 WHO Regional Office for Europe, Copenhagen, Denmark; 4 Department of Pulmonary Medicine, Bispebjerg Hospital, Copenhagen, Denmark; 5 WHO Collaborating Centre in Tuberculosis Laboratory Strengthening and the TB Supranational Reference Laboratory, San Raffaele Scientific Institute, Milan, Italy; 6 European Centre for Disease Prevention and Control, Stockholm, Sweden

**Keywords:** tuberculosis, infection, lung disease, HIV

## Abstract

**INTRODUCTION:**

Since 2015 (updated in 2019), the WHO has recommended to include the commercial lateral flow urine lipoarabinomannan TB test (LF-LAM), AlereLAM, in the diagnostic toolkit for severely ill people living with HIV.

**METHODS:**

To assess the current use and barriers to the implementation of the test, we conducted an electronic survey among national focal points and managers of TB and HIV programmes in the 53 Member States of the WHO European Region and a European network of clinicians working in TB and HIV medicine.

**RESULTS:**

In all, 45 individual responses (37 countries) were received from programme managers and focal points and 17 responses (14 countries) from clinicians. Only eight countries reported adopting LF-LAM policies, with only four currently using the AlereLAM (Armenia, Belarus, Ukraine and Uzbekistan). The most commonly reported barriers to implementing the test were the small number of eligible patients (with HIV-TB co-infections), the test not being included in the TB or HIV programme’s mandate and lack of budget allocation.

**CONCLUSION:**

Consistent with findings from high TB burden countries in Africa and Asia, the survey demonstrated that uptake of AlereLAM is almost non-existent. Addressing the identified barriers and the intrinsic limitations of the test could help to increase the use of the test.

## Abstract (fr)

**INTRODUCTION:**

Depuis 2015 (mise à jour en 2019), l’OMS recommande d’inclure l’AlereLAM, un test urinaire commercial de lipoarabinomannane à flux latéral (LF-LAM) parmi les outils de diagnostic pour le dépistage de la TB chez les personnes gravement malades vivant avec le VIH.

**MÉTHODES:**

Pour évaluer l’utilisation actuelle du test et les obstacles à sa mise en oeuvre, nous avons mené une enquête électronique auprès des points focaux nationaux et des responsables des programmes de lutte contre la TB et le VIH dans les 53 États membres de la Région européenne de l’OMS, ainsi qu’auprès d’un réseau européen de cliniciens travaillant dans le domaine de la TB et du VIH.

**RÉSULTATS:**

Au total, 45 réponses individuelles (37 pays) ont été reçues de la part de gestionnaires de programmes et de points focaux et 17 réponses (14 pays) de la part de cliniciens. Seuls huit pays ont déclaré avoir adopté des politiques relatives au LF-LAM, et quatre seulement utilisent actuellement l’AlereLAM (Arménie, Belarus, Ukraine et Ouzbékistan). Les obstacles les plus fréquemment signalés à la mise en oeuvre du test étaient le faible nombre de patients éligibles (co-infections VIH-TB), le fait que le test ne fasse pas partie du mandat du programme de lutte contre la TB ou le VIH et le manque d’allocation budgétaire.

**CONCLUSION:**

Conformément aux résultats obtenus dans les pays d’Afrique et d’Asie où la TB est très répandue, l’enquête a démontré que l’utilisation d’AlereLAM est presque inexistante. L’élimination des obstacles identifiés et des limites intrinsèques du test pourrait contribuer à accroître l’utilisation de ce dernier.

People living with human immunodeficiency virus (PLWH) are 20–30 times more likely than those without HIV to develop active TB, with TB the leading cause of death and hospitalization in PLWH.^1^,^2^ Although 37.2% of all deaths in PLWH are attributable to TB, TB remains undiagnosed at the time of death in 46% of cases.^3^ In the WHO European Region, approximately 246,000 people were diagnosed with TB in 2019; of these, 20,000 subsequently died, mostly those in Eastern Europe and Central Asia.^4^ Despite a fall in the average TB incidence in 2010–2019 by approximately 5% per year, the treatment success rate among all patients was only 77% in 2019, and WHO Regional TB-HIV coinfection rates increased from 7% to 12% between 2010 and 2019.^4^ In Eastern Europe 1-year mortality for PLWH with TB is 29%.^5^ In most settings, TB diagnosis relies on the detection of *Mycobacterium tuberculosis* in respiratory samples (e.g., sputum) using conventional smear microscopy and culture-based methods.^6^ TB diagnosis is often challenging among PLWH because they commonly present with extrapulmonary TB and have a low bacterial burden. Recently, polymerase chain reaction (PCR) rapid tests such as the Xpert^®^ MTB/RIF Ultra (Cepheid, Sunnyvale, CA, USA) have become available as an additional diagnostic tool for TB.^7^ Since 2015, the WHO has recommended the commercial point-of-care lateral flow urine lipoarabinomannan assay (LF-LAM), AlereLAM (Abbott Laboratories, Chicago, IL, USA), to improve TB diagnosis in severely ill PLWH.^8^–^10^ A systematic review and meta-analysis found a pooled sensitivity of 42% and a specificity of 91% for the test.^11^ The WHO currently recommends the AlereLAM test as part of the diagnostic toolkit for children, adolescents and adults living with HIV (both inpatients and outpatients) with the signs and symptoms of TB, with advanced HIV diseases and/or who are seriously ill with a CD4-cell count of <200 cells/mm^3^ (100 cells/mm^3^ for outpatients), irrespective of symptoms.^8^ However, uptake has been slow in many countries. A survey of 24 high TB-HIV burden African and Asian countries conducted before the 2019 update of the guidelines^12^ found that only 11 (46%) had adopted LF-LAM policies and only five (21%) were using AlereLAM.^13^ The uptake and potential barriers to LF-LAM use in Europe and central Asia have not been systematically assessed. To address this gap, a survey of national TB and HIV focal points, TB laboratory managers and clinicians working in TB-HIV was conducted to assess AlereLAM uptake and barriers in the WHO European Region.

## METHODS

A semi-structured electronic questionnaire was sent to 1) national focal points/managers of national TB programmes, 2) national focal points/managers of national HIV programmes, and 3) operational contact points for TB microbiology in all 53 Member States of the WHO European Region. In addition, a separate semi-structured questionnaire was emailed to an established research network of clinicians working in TB-HIV medicine in Europe.^14^ Both questionnaires were adapted from a previous survey,^13^ and updated to reflect the 2019 WHO guidelines on LF-LAM.^12^ Questionnaires in English (shown in Supplementary Data 1) or Russian were distributed using a network of national focal points from the European Centre for Disease Prevention and Control (ECDC) for the European Union (EU) and the European Economic Area (EEA) countries and via networks of the TB and HIV teams of the Division of Country Health Programmes, WHO Regional Office for Europe to TB-HIV programme managers in other countries (particularly those in Eastern Europe and Central Asia). These were also distributed through the ECDC European Reference Laboratory Network for Tuberculosis. Participants were invited to enter their responses directly into a specially designed REDCap database (Vanderbilt University, Nashville, TN, USA) between June and August 2021; in case of non-response, reminders were sent up to three times. As the questionnaire only retrieved national- or clinic-level data, ethical approval was not required. By completing the questionnaire, participants consented to the use of their de-identified data for research purposes.

Responses were summarised in a descriptive way, as reported previously by Singhroy et al.^13^ Where responses were received from multiple participants in the same country, the most complete response for policy adoption and the AlereLAM use algorithm were recorded, along with any disagreement between responses. All responses about barriers to implementation were recorded independently.

## RESULTS

**Figure 1:**
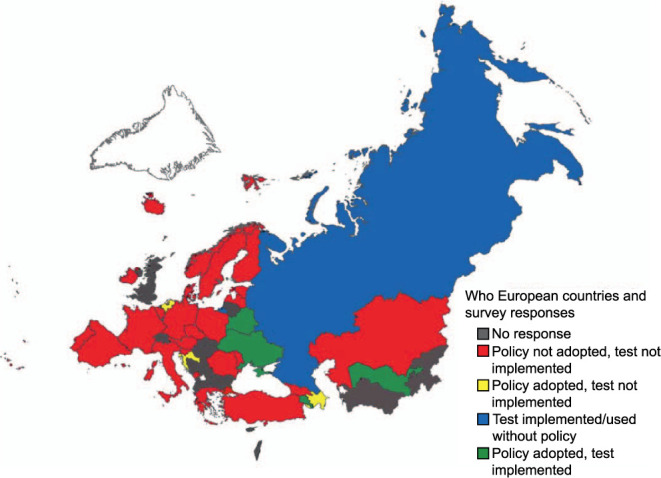
WHO European Region countries contacted for the survey.

Relevant individuals were contacted in all 53 countries of the WHO European Region, with 45 complete responses received from participants in 37 countries (Figure 1). Of these, 18 (40%) were received from national TB focal points (the EU and EEA countries) or TB programme managers (non-EU countries) and 11 (24%) came from operational contact points for TB microbiology, 3 (7%) from national focal points for HIV (ECDC) and 13 (29%) from other national contacts in ECDC and the WHO Regional Office for Europe networks. In addition, of the 30 clinicians contacted, 17 responses were received from clinicians working with HIV and/or TB in 14 individual countries.

### Data from focal points, TB-HIV programme managers and operational contact points for TB microbiology

At least one respondent from most countries with a response (27/37, 73%) was familiar with both WHO guidelines (2015 and 2019 versions) recommending AlereLAM or with either the 2015^9^ or the 2019^8^ version (one respondent for each; Supplementary Table S1). Respondents from nine of the 37 countries were unfamiliar with both documents (24%) (one country information not available).

Of the 37 countries with responses, only 7 (19%) had policies that included AlereLAM (Supplementary Table S1): 3 included AlereLAM in both the national TB and HIV programmes, 2 in the national TB programme only and 2 in the national HIV programme only. Of the 7 countries with policies, only 4 had implemented AlereLAM, and 1/4 that had implemented the test, was using the test only at a single pilot hospital. In addition, one country with no policy on AlereLAM was using the test as a private out-of-pocket option for patients.

Of the 30 countries with no policy on AlereLAM and/or not currently implementing the test, 7 countries (23%) were planning to use AlereLAM in the near future (2021–2023), respondents in 15 (50%) did not know whether there were plans to use the test in the future and 9 (30%) did not plan to use the test in the future. Of the 9 countries that either had policies including AlereLAM (*n* = 8) or were using the test without having a policy (*n* = 1), respondents from 5 provided information on the current algorithm used for AlereLAM testing (Table). Only three countries (Armenia, Belarus and Ukraine) were using the AlereLAM test in accordance with current WHO guidelines (2019 version). The Uzbek policy documents did not specify an algorithm for applying the test; however, the test was mainly used in accordance with current WHO guidelines. The Russian Federation had no official policy including AlereLAM.

**Table:** AlereLAM testing: existing algorithms (recommendations) in policy documents (or guidelines) in WHO European Region countries having responded to have policies on AlereLAM or are using the test ^*^

AlereLAM algorithm/location of use	Armenia	Azerbaijan	Belarus	Croatia	Netherlands	Russian Federation	Ukraine	Uzbekistan
Use of current algorithm for AlereLAM testing in adults								
To assist in the diagnosis of TB in adult HIV-positive inpatients with signs and symptoms of TB (PTB and/or EPTB)	Yes	Not available	Yes	Not available	Not available	Yes	Yes	Yes^†^
To assist in the diagnosis of TB in adult HIV-positive inpatients with advanced HIV disease or who are seriously ill	Yes	Not available	Yes	Not available	Not available	–	Yes	Yes^†^
To assist in the diagnosis of TB in adult HIV-positive inpatients irrespective of signs and symptoms of TB and with a CD4-cell count <200 cells/mm^3^	Yes	Not available	Yes	Not available	Not available	–	Yes	Yes^†^
To assist in the diagnosis of TB in adult HIV-positive outpatients (ambulatory) with signs and symptoms of TB (PTB and/or EPTB) or seriously ill	Yes	Not available	Yes	Not available	Not available	Yes	Yes	Yes^†^
To assist in the diagnosis of TB in adult HIV-positive outpatients (ambulatory) irrespective of signs and symptoms of TB and with a CD4-cell count <100 cells/mm^3^	Yes	Not available	Yes	Not available	Not available	–	Yes	Yes^†^
To assist in the diagnosis of TB in adult HIV-positive outpatients (ambulatory) without assessing TB symptoms	–	Not available	–	Not available	Not available	–	–	–
Other	–	–	–	–	–	–	–	–
Use of current algorithm for AlereLAM testing in children/adolescents								
To assist in the diagnosis of TB in children/adolescent HIV-positive inpatients with signs and symptoms of TB (PTB and/or EPTB)	Yes	Not available	Yes	Not available	Not available	–	Yes	Yes^†^
To assist in the diagnosis of TB in children/adolescent HIV-positive inpatients with advanced HIV disease or who are seriously ill	Yes	Not available	Yes	Not available	Not available	–	Yes	Yes^†^
To assist in the diagnosis of TB in children/adolescent HIV-positive inpatients irrespective of signs and symptoms of TB and with a CD4-cell count of <200 cells/mm^3^	Yes	Not available	Yes	Not available	Not available	–	Yes	Yes^†^
To assist in the diagnosis of TB in children/adolescent HIV-positive outpatients (ambulatory) with signs and symptoms of TB (PTB and/or EPTB) or seriously ill	Yes	Not available	Yes	Not available	Not available	–	Yes	Yes^†^
To assist in the diagnosis of TB in children/adolescent HIV-positive outpatients (ambulatory) irrespective of signs and symptoms of TB and with a CD4-cell count of <100 cells/mm^3^	Yes	Not available	Yes	Not available	Not available	–	Yes	Yes^†^
To assist in the diagnosis of TB in children/adolescent HIV-positive outpatients (ambulatory) without assessing TB symptoms	–	Not available		Not available	Not available	–	–	–
Other	–	–	–	–	–	–	–	–
Where is the AlereLAM test being used?								
All hospitals	Yes	–	–	–	–	–	–	–
Limited number of hospitals	–	–	Yes	–	–	Yes	Yes^‡^	–
All ART centres	–	–	–	–	–	–	–	–
Limited number of ART centres	–	–	–	–	–	–	–	Yes
Other	–	–	–	–	–	–	–	–

^*^ Countries with policies that include AlereLAM testing, *n* = 8; countries using AlereLAM testing, *n* = 1.

^†^ To date, the country’s policy documents do not specify a specific algorithm for applying this test.

^‡^ Several contradictory answers were given.

LAM = lipoaribomannan; PTB = pulmonary TB; EPTB = extrapulmonary TB; ART =antiretroviral therapy.

Armenia was the only country in which the test was reported to be available at all hospitals. In Belarus, the Russian Federation and Ukraine, the test was reported to be available at a limited number of hospitals, in the Russian Federation, only as a private out-of-pocket option; and in Belarus, only at the one centre in the capital, Minsk. In Uzbekistan, the test was reported to be available at a limited number of antiretroviral therapy (ART) centres. A respondent from Azerbaijan reported that it was planned to be included in the next TB-HIV project of the Global Fund to Fight AIDS, Tuberculosis and Malaria as an operational research component in penitentiary institutions. The respondent from Georgia reported that the country is planning to negotiate the national HIV programme and to include AlereLAM testing in the 2023–2025 funding request to the Global Fund. The test was unavailable in all of the other 31 countries.

**Figure 2:**
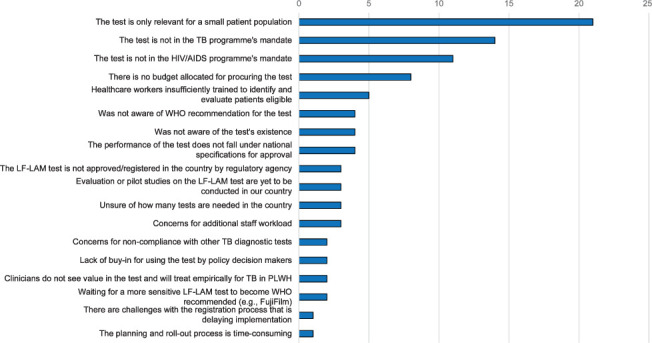
Barriers to AlereLAM urine test use a reported by TB and HIV focal points and programme managers in the WHO European Region. If respondents from the same country reported the same barrier, it was only recorded once. LF-LAM = lateral flow urine lipoaribomannan; PLWH = people living with HIV; FujiLAM = Fujifilm SILVAMP TB LAM assay.

In most of the 37 countries (*n* = 28, 76%), respondents identified barriers preventing the implementation of AlereLAM testing (Figure 2). The most commonly reported barrier, particularly in Western and Central Europe, was that AlereLAM testing is relevant to relatively few patients in the country and, therefore, its implementation was not a priority (21/28, 75%), followed by the test not being included in the mandate of the TB programme (14/28, 50%) or HIV/AIDS programme (11/28, 39%) and no budget allocation for procuring the test (8/28, 29%). A respondent from France reported that she was interested in implementing the AlereLAM and had attempted for the last 5 years to buy it, but that the producing company had been unable to sell the test in France owing to a lack of regulatory approval (e.g., CE marking).^*^

### Data from clinicians working with HIV and/or TB patients

Of the 17 clinicians from 14 countries who responded to the survey, most (*n* = 12, 71%) were familiar with both the 2015 and 2019 versions of the WHO guidelines on lateral flow urine LAM testing, 2 (12%) were aware of only 2015 version and 2 (12%) of only the 2019 version (Supplementary Table S2). Only one (6%) did not know about either version of the WHO guidelines. Three clinicians (18%) (from Minsk, Belarus; Tbilisi, Georgia; and Kharkov, Ukraine) reported using the test in their department (all three worked in HIV clinics). However, the test is not currently available in the Georgian clinic as it is waiting for a more sensitive LAM test (e.g., Fujifilm-LAM; Fujifilm, Tokyo, Japan) to become available. Two TB clinics (12%) (one in Minsk, Belarus; and the other in St Petersburg, the Russian Federation) are planning to introduce the test in the near future. Respondents from Belgium, Denmark and the United Kingdom were interested in using the test but said that they could not obtain it.

**Figure 3:**
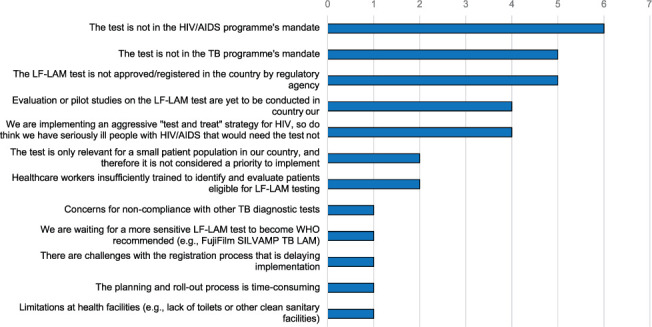
Clinicians: barriers to AlereLAM test use. Unique barriers per country reported. If two respondents from the same country reported the same barrier, it was only reported once. LF-LAM = lateral flow urine lipoaribomannan.

Clinicians from all 14 countries except for Belarus (93%) reported barriers to implementing and using the test (Figure 3). Respondents from six countries (43%) reported the test was not included in the HIV/AIDS programme’s or TB programme’s mandate, and another five (36%) that it was not approved by the country’s regulatory authority.

## DISCUSSION

In agreement with a previous survey of high TB burden countries in Asia and Africa, this study shows that the implementation of AlereLAM testing remains low in the WHO European Region, despite widespread knowledge of WHO guidelines recommending implementation of the test.^13^ Furthermore, policy adoption was lower (19%) in the present survey than in the previous one (46%).^13^ Although the present survey found that seven countries have policies recommending use of the test, clinical implementation is only taking place in four, and in some of these its use is limited to pilot projects or individual healthcare centres/laboratories. Responses were consistent between focal points/programme managers and clinicians. In most countries that are implementing AlereLAM (Armenia, Belarus, Ukraine), WHO guidelines and recommendations are generally being followed.

The most commonly reported barrier to incorporating AlereLAM testing into national recommendations, programmes and clinics was that the test is only relevant to a small proportion of patients. Although some clinicians in Western European countries were interested in obtaining and implementing AlereLAM, its availability was hindered by a lack of regulatory approval and the reluctance of the producer to market it in the countries. This is unfortunate because even where the prevalence of TB-HIV coinfection is low, AlereLAM testing can be a helpful diagnostic tool. It has a rapid turnaround time of less than an hour from hospital admission, reduces mortality and is particularly useful for testing those without pulmonary TB or those unable to produce sputum.^15^,^16^ Very few programme managers (*n* = 2) and clinicians (*n* = 0) reported concerns of additional staff workload and non-compliance with other TB tests.

Respondents from seven countries, mostly in Eastern Europe and Central Asia, mentioned a lack of budget allocation as an implementation barrier. This is an important signal to donor agencies (e.g., the Global Fund) to prioritise funding for the test. It also requires awareness and knowledge among policy makers and clinicians to advocate for increased targeted funding for TB diagnostics in general (including GeneXpert platforms and drug-susceptibility testing) among domestic governments.

Other prominent barriers such as the lack of awareness that the test exists and of the WHO guidelines, as well as a lack of training for healthcare workers on using the test can be overcome by improved educational efforts of regional and national public health institutions. In particular, the improved evidence included in the latest 2019 guidelines (which include broader, more general WHO recommendations with less emphasis on CD4 cell counts) might help to increase the confidence of policy makers and clinicians in recommending the test.^8^

Among clinicians, the most commonly reported barrier (besides lack of recommendations in the TB-HIV programme) was the absence of approval from regulatory agencies. This might be due to the manufacturer giving low priority to seeking approval owing to a perceived lack of demand/small market size in the WHO European Region. However, responses from a number of clinicians and the results of the previous meta-analysis demonstrate that the AlereLAM test could be a useful addition to the TB diagnostic package for PLWH. Centralised approval from European institutions could facilitate access to the test in EU countries. In other countries of the WHO European Region, funders and public health authorities could assist the manufacturer to obtain regulatory approval.

The survey obtained responses from focal points or programme managers from 37 of the 53 countries in the WHO European Region. Unfortunately, there was no response from 16 countries, including some large countries (e.g., the United Kingdom), and some with a high HIV and/or TB burden (e.g., Lithuania, Tajikistan and Turkmenistan). It is likely that the countries without a reply do not have policies including AlereLAM and are not implementing the test – and therefore are be less inclined to respond to the survey. Disproportionately more TB programme managers and focal points responded, with only three responses from HIV programme managers. Both could lead to underestimation of the degree of implementation, in particular if the test is being predominantly implemented through HIV programmes. Furthermore, responses were received from only 17 clinicians, which precludes a more general assessment of congruence between official policy and clinical implementation.

Despite these limitations, this is the first study to provide a comprehensive overview of policy and implementation of AlereLAM urine point-of-care testing in the WHO European Region. Both programme managers and clinicians in all parts of the Region expressed interest in implementing and using the test. In many countries of Eastern Europe and Central Asia, the test could form an essential part of a comprehensive diagnostic package for PLWH, and in other parts of the Region it could be a valuable addition to current clinical practice. Although the availability of TB diagnostics and treatment has significantly improved in Eastern Europe in the last decade, there is still an unmet need to ensure that molecular diagnostics (e.g. Cepheid’s Xpert^®^ MTB/RIF) and drug susceptibility testing are available at all relevant hospitals and clinics.^17^ The barriers identified in this study could guide policy makers, national governments, clinicians, scientists and donors in improving access to the AlereLAM test in all countries in the Region. Some of these barriers, such as the lack of approval from regulatory agencies, should be relatively easy to overcome. Future efforts should focus on increasing the number of science-based pilot projects, including implementation of the Fujifilm-LAM test, which has better sensitivity and specificity (independent of CD4 cell counts), and educating policy makers and clinicians about the existing evidence, WHO recommendations and advantages of AlereLAM testing.^18^

## Footnotes

^*^ CE marking indicates that a product meets EU safety, health and environmental protection requirements and can be marketed in the EU.

## References

[1] Kwan CK, Ernst JD (2011). HIV and tuberculosis: a deadly human syndemic. Clin Microbiol Rev.

[2] Ford N (2016). TB as a cause of hospitalization and in-hospital mortality among people living with HIV worldwide: a systematic review and meta-analysis. J Int AIDS Soc.

[3] Gupta RK (2015). Prevalence of tuberculosis in post-mortem studies of HIV-infected adults and children in resource-limited settings: a systematic review and meta-analysis. AIDS.

[4] European Centre for Disease Prevention and Control (2021). Tuberculosis surveillance and monitoring in Europe 2021–2019 data. https://www.ecdc.europa.eu/en/publications-data/tuberculosis-surveillance-and-monitoring-europe-2021-2019-data.

[5] Podlekareva DN (2016). Tuberculosis-related mortality in people living with HIV in Europe and Latin America: an international cohort study. Lancet HIV.

[6] Getahun H (2007). Diagnosis of smear-negative pulmonary tuberculosis in people with HIV infection or AIDS in resource-constrained settings: informing urgent policy changes. Lancet.

[7] Dorman SE (2018). Xpert MTB/RIF Ultra for detection of *Mycobacterium tuberculosis* and rifampicin resistance: a prospective multicentre diagnostic accuracy study. Lancet Infect Dis.

[8] World Health Organization Consolidated guidelines on tuberculosis. Module 2: Screening – Systematic screening for tuberculosis disease. https://www.who.int/publications/i/item/9789240022676.

[9] World Health Organization (2015). The use of lateral flow urine lipoarabinomannan assay (LF-LAM) for the diagnosis and screening of active tuberculosis in people living with HIV: policy guidance.

[10] Correia-Neves M (2019). Biomarkers for tuberculosis: the case for lipoarabinomannan. ERJ Open Res.

[11] Bjerrum S (2019). Lateral flow urine lipoarabinomannan assay for detecting active tuberculosis in people living with HIV. Cochrane Database Syst Rev.

[12] World Health Organization (2019). Lateral flow urine lipoarabinomannan assay (LF-LAM) for the diagnosis of active tuberculosis in people living with HIV. Policy update, 2019.

[13] Singhroy DN (2020). Adoption and uptake of the lateral flow urine LAM test in countries with high tuberculosis and HIV/AIDS burden: current landscape and barriers. Gates Open Res.

[14] Centre of Excellence for Health, Immunity and Infections (2011). TB:HIV Study Group. https://chip.dk/Research/Studies/TBHIV/TBHIV-Study-Group.

[15] Lawn SD (2017). Diagnostic accuracy, incremental yield and prognostic value of Determine TB-LAM for routine diagnostic testing for tuberculosis in HIV-infected patients requiring acute hospital admission in South Africa: a prospective cohort. BMC Med.

[16] Nathavitharana RR (2021). Impact of diagnostic strategies for tuberculosis using lateral flow urine lipoarabinomannan assay in people living with HIV. Cochrane Database Syst Rev.

[17] Bentzon AK (2021). Healthcare delivery for HIV-positive people with tuberculosis in Europe. HIV Med.

[18] Broger T (2019). Novel lipoarabinomannan point-of-care tuberculosis test for people with HIV: a diagnostic accuracy study. Lancet Infect Dis.

